# A cross-sectional study confirms temporary post-COVID-19 vaccine menstrual irregularity and the associated physiological changes among vaccinated women in Jordan

**DOI:** 10.3389/fmed.2023.1211283

**Published:** 2023-10-06

**Authors:** Ensaf Y. Almomani, Rima Hajjo, Ahmad Qablan, Dima A. Sabbah, Abass Al-Momany

**Affiliations:** ^1^Department of Basic Medical Sciences, Faculty of Medicine, Al-Balqa Applied University, Al-Salt, Jordan; ^2^Applied Science Research Center, Applied Science Private University, Amman, Jordan; ^3^Department of Pharmacy, Faculty of Pharmacy, Al-Zaytoonah University of Jordan, Amman, Jordan; ^4^Laboratory for Molecular Modeling, Division of Chemical Biology and Medicinal Chemistry, Eshelman School of Pharmacy, The University of North Carolina at Chapel Hill, Chapel Hill, NC, United States; ^5^Jordan CDC, Amman, Jordan; ^6^Department of Curriculum and Methods of Instruction, United Arab Emirates University, Al Ain, United Arab Emirates; ^7^Faculty of Educational Sciences, Hashemite University, Zarqa, Jordan; ^8^Department of Clinical Laboratory Sciences, University of Jordan, Amman, Jordan

**Keywords:** COVID-19 vaccine, adverse events, menstrual cycle irregularity, vaccinated women, Jordan

## Abstract

**Background:**

COVID-19 vaccines continue to save people’s lives around the world; however, some vaccine adverse events have been a major concern which slowed down vaccination campaigns. Anecdotal evidence pointed to the vaccine effect on menstruation but evidence from the adverse event reporting systems and the biomedical literature was lacking. This study aimed to investigate the physiological changes in women during menstruation amid the COVID-19 vaccination.

**Methods:**

A cross-sectional online survey was distributed to COVID-19 vaccinated women from Nov 2021 to Jan 2022. The results were analyzed using the SPSS software.

**Results:**

Among the 564 vaccinated women, 52% experienced significant menstrual irregularities post-vaccination compared to before regardless of the vaccine type. The kind of menstrual irregularity varied among the vaccinated women, for example, 33% had earlier menstruation, while 35% reported delayed menstruation. About 31% experienced heavier menstruation, whereas 24% had lighter menstrual flow. About 29% had menstruation last longer, but 13% had it shorter than usual. Noteworthy, the menstrual irregularities were more frequent after the second vaccine shot, and they disappeared within 3 months on average. Interestingly, 24% of the vaccinated women reported these irregularities to their gynecologist.

**Conclusion:**

The COVID-19 vaccine may cause physiological disturbances during menstruation. Luckily, these irregularities were short-termed and should not be a reason for vaccine hesitancy in women. Further studies are encouraged to unravel the COVID-19 vaccine adverse effect on women’s health.

## Introduction

COVID-19 vaccines have decreased the burden of SARS-CoV-2 infections worldwide by averting 80–90% of hospitalized individuals from getting reinfected and attenuating 40–65% of signs and symptoms (1, 2). The availability of various vaccine platforms has saved people’s lives and reduced hospital care and death toll, distinctly through the first 6 months of being fully vaccinated with two doses (3–6). Vaccines have circumvented the prevalence of COVID-19, prevented severe pandemic waves in vaccinated communities, and decreased lockdown protocols and governmental inspection measures (7, 8). The widely distributed vaccines platforms that were approved by either the US Food and Drug Administration (FDA) or the World Health Organization (WHO) were: messenger RNA (mRNA) vaccines, viral vector (adenovirus) vaccines, and inactivated (killed SARS-CoV-2 virus) vaccines.

While COVID-19 vaccines were important tools in the fight against SARS-CoV-2, they were associated with adverse events like any other pharmaceutical drugs. Some adverse events were common or frequent, and others were rare or uncommon. For example, the most frequently reported COVID-19 vaccine adverse events were headache, fever, fatigue and muscle pain. BioNTech/Pfizer vaccine triggered diverse adverse effects within 27% of vaccinated people (9) including arrhythmia, fatigue, headache, lymphadenopathy, right leg paresthesia, and vaccination site injury (5, 10, 11). Moderna’s vaccine resulted in chills, fatigue, headache, joint and muscle pain, rashes, and pain or swelling at the injection site (12). Johnson & Johnson’s vaccine led to blood clots in vaccinated people (13). Sinopharm’s vaccine was accompanied with fatigue, lethargy, headache, and tenderness especially after the second vaccine shot, and in higher frequency in females compared to males (14).

Adverse event databases as the vaccine adverse event reporting system (VAERS) and the Eudra Vigilance European database, highlighted serious cardiovascular adverse events after receiving COVID-19 vaccines including myocarditis, pericarditis, venous thromboembolism, hemorrhage, myocardial infarction, and stroke (15). In fact, many published articles reported on the development of acute myocarditis and pericarditis following COVID19 vaccination (16–18) and derived mechanistic hypotheses to explain these events (16, 17, 19).

Fears that COVID-19 vaccines could affect the female reproductive system escalated both on social media and in the scientific literature. Menstrual adverse events including menstrual cycle irregularity were reported after receiving COVID-19 vaccines (20). Studies reported that 50–60% of reproductive age women, who received the first dose of COVID-19 vaccine, have menstrual cycle disturbances regardless of the administered vaccine platforms (21). Changes in periodicity of the succeeding menstrual cycles were reported after the first vaccination dose of the different vaccine types; 66.7% of AstraZeneca (Vaxzevria), 57.1% of Pfizer-BioNTech (Comirnaty), 47.4% of Moderna (Spikevax), and 33.3% of Johnson & Johnson (Janssen) vaccinated females (21). The prevalent menstrual changes were longer and heavier menstrual cycles than normal (21) in addition to a minor change in the cycle length (22). It should be noted that COVID-19 infection itself affected women’s reproductive system and led to menstrual cycle changes. A clinical study on women of reproductive age showed that 25% of COVID-19 infected females had menstrual disturbances (23) and they experienced changes in mensural volume and/or cycle duration after being diagnosed with COVID-19 infection (23). In addition to SARS-CoV-2 infection itself and COVID-19 vaccines, stress was suggested as a plausible reason for menstrual cycle changes during the pandemic (24, 25).

Although, the UK’s Medicines and Healthcare products Regulatory Agency (MHRA) (20) did not confirm a potential link between menstrual changes and COVID-19 vaccines (26, 27). The agency recommended further probing of post-COVID-19-vaccine menstrual disturbances (26, 28, 29). However, post-vaccine menstrual changes were not frequently reported in vaccine adverse effects reporting systems including VAERS database of the United States (26, 27) and local healthcare records from Jordan. We attributed this to that fact that women do not often perceive menstrual changes as an immediate threat to their lives and therefore may not seek medical attention or report it to healthcare authorities. Additionally, the vaccine effect on menstruation was understudied in Jordan and the Middle East, despite an increasing number of reports circulating on social media regarding post-vaccine adverse effects on the menstrual cycle and fertility, leading to more vaccine-hesitancy among Jordanian women. Thus, we opted to investigate the association between COVID-19 vaccines and menstrual changes and study the physiological changes in women during menstruation amid after receiving COVID-19 vaccine.

The rationale for conducting this study relied on increasing number of reports in the biomedical literature pointing to a possible relationship between COVID-19 vaccines and menstrual changes. These reports were exploited by anti-vaxxers who claimed that vaccine-induced menstrual changes could cause infertility. This led to increased rates of vaccine hesitancy among women world-wide including women in Middle Eastern countries like Jordan where people prefer large families and put more emphasis on fertility issues.

The main questions that this study aimed to answer were the following:

1. Are COVID-19 vaccines associated with menstrual cycle irregularities among Jordanian women?
2. Do post-vaccine menstrual cycle irregularities depend on the type of vaccine?
3. Does the COVID-19 vaccine type affect the duration of menstrual cycle irregularities?

## Methods

### Study design

This was an anonymous observational cross-sectional study. Quantitative data methodology was used to collect data. A specifically designed questionnaire was used to collect data from participants who were COVID-19 vaccinated women.

### Data collection and data processing

The vaccinated women questionnaire was prepared and edited by the research team which includes physiologists, medical pharmacists, and a professor in the Department of Biology Education. The development of the questionnaire was guided by the one that Phelan et al. (30) used in their study.

### Study validation

For validation, initial drafts of the questionnaire were submitted to a group of experts to comment on the items’ relevance, clarity, and language accuracy. The reviewers were asked to suggest any additions, deletions, or replacements of any unsuitable items in the questionnaires. Based on the experts’ feedback, some modifications were introduced to the questionnaire to make it ready for the administration stage. The study was conducted and reported according to published best practice guidelines for reporting observational studies (STROBE) (31).

The validated version of the questionnaire consisted of three parts: Part 1: discussed the socio-demographic information of the vaccinated women including (age, marital status, functional status, educational level, and place of living). Part 2: collected COVID-19 vaccine information from the vaccinated women (the type of vaccine and the number of vaccine shots). Part 3: discussed the period status of the vaccinated women, it included two sections, 3-A: included factors that may cause menstrual cycle irregularities among the vaccinated females such as using contraceptives, and the presence of any of the common female reproductive problems including; ovarian cysts, pelvic inflammatory diseases, uterine fibroids, thyroid disorders, and osteoporosis. 3-B: evaluated the menstrual cycle irregularities details like the first incidence, quality, and duration.

The study’s questionnaire was included into an online survey which had an introductory paragraph describing the aim of the study. Additionally, it was mentioned in the survey that the participation in this study is voluntary and optional, and that the participants’ information is highly confidential. Filling the survey was estimated to take 3–5 min to finish. The survey was prepared in English, then translated to Arabic and was distributed in Arabic language. Survey responses were translated back to English. All translations were made using the forward-backward translation techniques (32). A full list of survey questions can be found in Supplementary Tables 1, 2.

The survey was published from November 2021 through January 2022 using Google application forms, and the survey link was distributed via email as well as social media channels including Facebook and WhatsApp applications. Ethical approval was granted for the study by the Al-Zaytoonah University Ethics Unit. Written consent was not required as all data was collected anonymously.

The survey was distributed by the research team to eligible individuals who were females with ages 16 and above, i.e., all women who were eligible to take the COVID-19 vaccine according to the Ministry of Health in Jordan.^1^ Eligibility criteria included the following: healthy, non-pregnant, non-lactating women, with no restrictions on the demographic information. The required sample size was calculated using the online Raosoft sample size calculator.^2^ The target study population consisted of the fully vaccinated population in Jordan as of November 1st, 2021 was 3,534,187 (see text footnote 1). Ideally, the percentage of vaccinated women should have been considered as the target population, but this information was not publicly available. That said, the calculated minimum sample size was 385, at a confidence level of 95%. A sample size calculation was performed. The actual sample size was 564 considering all vaccinated women who answered the survey questions, with a margin of error of 4.13%.

### Statistical analysis

Raw data were coded and analyzed using IBM’s SPSS software (2020) (SPSS Inc., Chicago, IL, United States). Parametric data is reported as mean and standard deviation (SD). Chi-Square test was used to analyze non-Parametric data. Descriptive and analytical statistical measures and indices were calculated to answer the proposed research questions. Pearson correlation coefficients were used to assess the strength of associations between vaccines and several menstrual cycle irregularities among the vaccinated women. A *p*-value ≤ 0.05 was considered statistically significant.

### Searching the vaccine adverse event reporting system (VAERS)

The vaccine adverse event reporting system (VAERS) (33) database was searched for “menstrual irregularity” adverse events in response to COVID-19 vaccines. Our query consisted of searching “all symptoms” for “COVID-19 vaccines” included in the database. Filtering was later done on “irregularity menstrual,” and manually inspecting all terms linked to menstruation.

## Results

### The sociodemographic information of the vaccinated women

**Figure 1:**
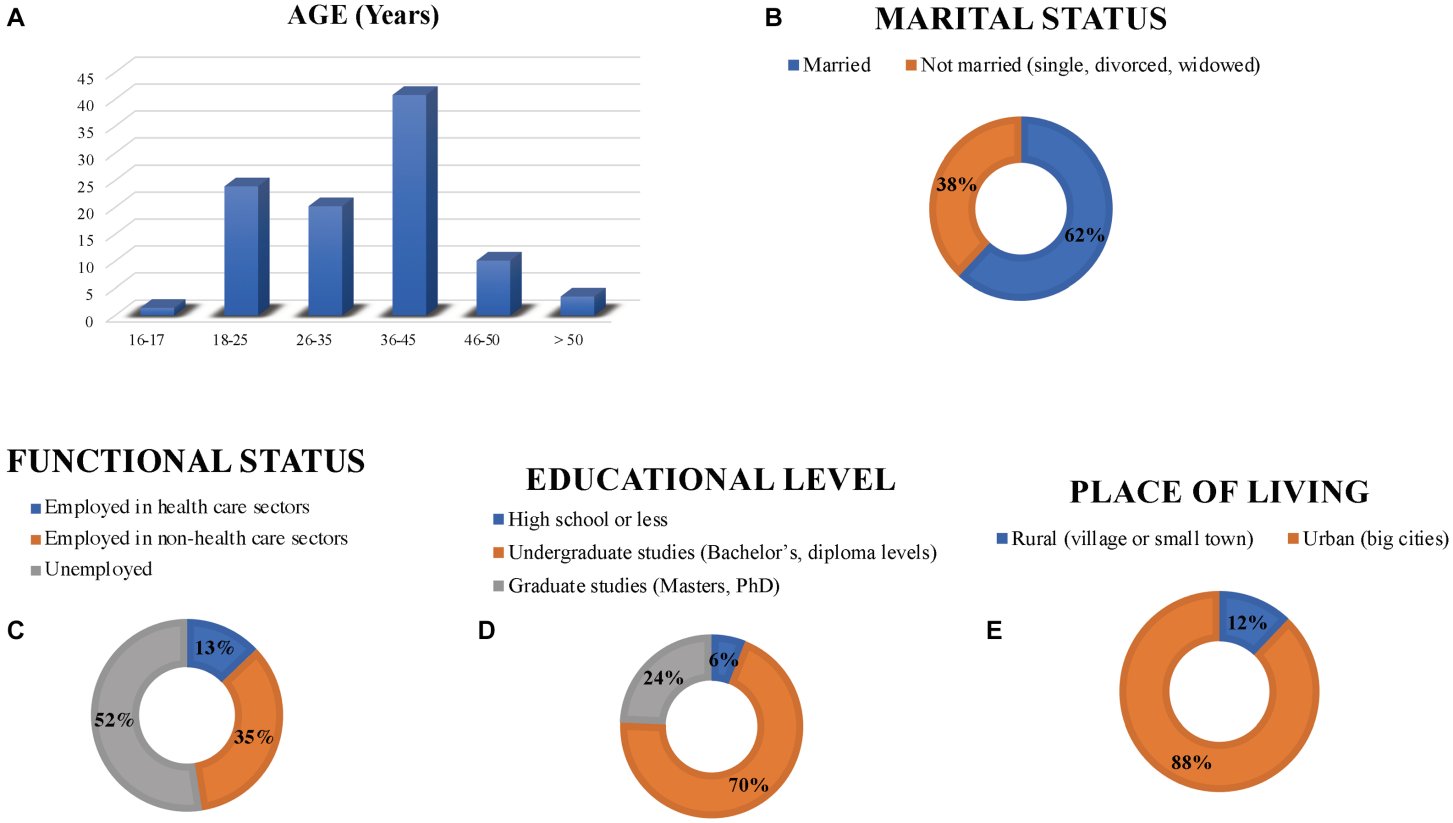
The sociodemographic information of the vaccinated women. **(A)** A bar chart represents the vaccinated women’ age categories in years. **(B)** A doughnut chart represents the vaccinated women marital status. **(C)** A doughnut chart represents vaccinated women functional status. **(D)** A doughnut chart represents the vaccinated women educational level. **(E)** A doughnut chart represents the vaccinated women place of living.

The sociodemographic information of the 564 vaccinated women was summarized in Figure 1. Eighty-five percent of the vaccinated women were between 18 and 45 years old, 62% of them were married. Regarding the employment status, 48% were employed, 12.9% of them were employed in health care sectors. Concerning the educational level, about 70% had either Diploma or Bachelor’s degree. A large share (87.9%) of the surveyors were living in urban areas.

### The COVID-19 information of the vaccinated women

**Figure 2:**
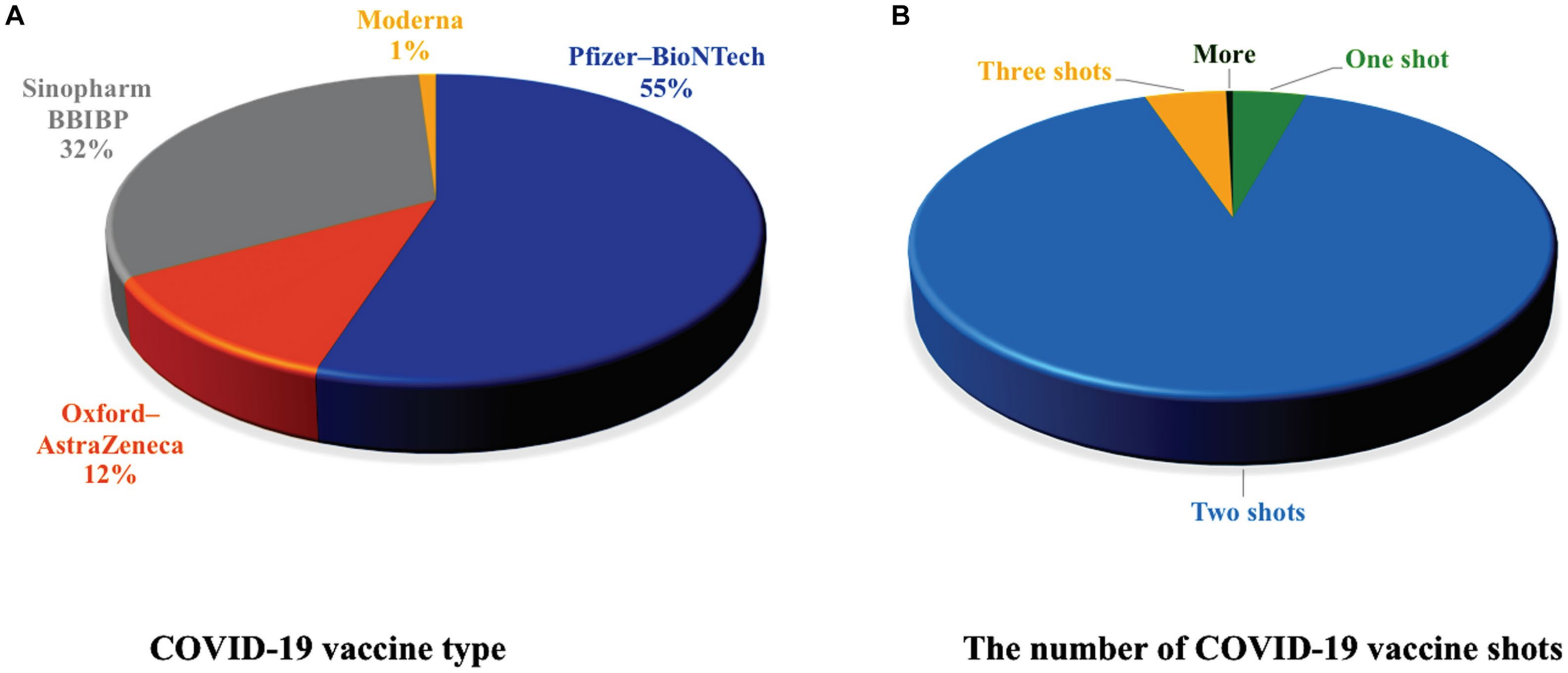
The COVID-19 information for the vaccinated women. **(A)** A pie chart showing the percentage of the different types of COVID-19 vaccines used by the vaccinated women. 55% had Pfizer–BioNTech, 12.0% Oxford–AstraZeneca, 32.0% Sinopharm BBIBP, and 1.0% had Moderna. **(B)** A pie chart representing the number of vaccine shots received by the vaccinated women. Most of the women received 2 shots of the COVID-19 vaccine.

The vaccine information for participating women was shown in Figure 2. About 55% of the surveyed women had Pfizer–BioNTech, 12.0% Oxford–AstraZeneca, 32.0% Sinopharm BBIBP, and only 1.0% had Moderna vaccine (Figure 2A). Most of the vaccinated women (90.6%) had two vaccine shots of the same vaccine type (Figure 2B). Noteworthy, the third vaccine shot that was given to people in Jordan was only Pfizer–BioNTech vaccine.

### The vaccinated women health baseline characteristics before and after COVID-19 vaccination

To assess the effects of the COVID-19 vaccines on women’s menstrual cycles, the surveyed women answered questions about their menstruation status before and after vaccination (Table 1). About 18% of the study participants had menstrual cycle irregularities before vaccination, whereas 52% reported post-vaccine menstrual irregularities. Of note, less than 20% of the participants were using contraceptives before and after vaccination (Table 1). The vaccinated women also reported any health issues they had that may affect menstruation. Results in Table 1 showed that 10.5% of vaccinated women had ovarian cysts, 3.2% had pelvic inflammatory disease, 3.7% had uterine fibroids, 8.3% had thyroid disorders, and only 2% had osteoporosis (Table 1).

**Table 1:** The vaccinated women health baseline characteristics before and after COVID-19 vaccination.

	Percentage (%)	Pearson correlation (*r*)	Did you have menstrual irregularity before COVID-19 vaccination?	Did you experience menstrual irregularity after vaccination?
**Before COVID-19 vaccination**				
Did you have menstrual irregularity?		*r*	1	0.199^*^
Yes	18.3	*p*-value		0.000
No	81.7	N	564	564
Were you using contraceptives?		*r*	0.009	0.004
Yes	19.1	*p*-value	0.841	0.931
No	80.9	N	550	550
Did you have ovarian cysts?		*r*	0.183^**^	0.075
Yes	10.5	*p*-value	0.000	0.076
No	89.5	N	564	564
Did you have pelvic inflammatory disease?		*r*	0.019	0.115^*^
Yes	3.2	*p*-value	0.659	0.006
No	96.8	N	564	564
Did you have uterine fibroids?		*r*	−0.044	0.059
Yes	3.7	*p*-value	0.292	0.164
No	96.3	N	564	564
Did you have thyroid disorders?		*r*	0.073	0.073
Yes	8.3	*p*-value	0.082	0.084
No	91.7	N	564	564
Do you have osteoporosis?		*r*	0.033	0.033
Yes	2.0	*p*-value	0.436	0.427
No	98.0	N	564	564
After COVID-19 vaccination				
Were you using contraceptives?		*r*	0.003	0.019
Yes	18.6	*p*-value	0.947	0.653
No	81.4	N	549	549
Did you experience menstrual irregularity		*r*	0.199^*^	1
Yes	51.8	*p*-value	0.000	
No	48.2	N	564	564

* Correlation is significant at the 0.01 level (two-tailed), r: Pearson Correlation Coefficient; *p*- value: the significance level; N: number of vaccinated women.

### Menstrual cycle irregularity description

Most of the vaccinated women (65%) noticed menstrual irregularities after the second vaccine shot (Table 2). Women had distinct responses describing the nature of menstrual irregularities. About 33% of women reported shorter menstrual cycle than usual (less than 21 days), whereas 35% experienced longer menstruation than usual (more than 35 days). 8.8% of women missed at least one period. About 31% of women had heavier menstrual flow than normal, while 23.8% had it lighter. 29% of the vaccinated women had menstruation lasted longer than usual, but 12.8% had it shorter. 20% of women reported a menstruation accompanied with pain, cramping, and nausea or vomiting. About 11% had intermenstrual bleeding.

**Table 2:** Menstrual cycle irregularity description.

	Questions	Number	Percentage %
1	When did you notice the changes in period? (290 response)		
	After the first vaccination shot	102	35.2
	After the second vaccination shot	187	64.5
	After the third vaccination shot	1	0.3
2	Describe the kind of irregularity you had by choosing from the following (320 response)		
	Periods that occur less than 21 days	107	33.4
	Periods that occur more than 35 days apart	112	35
	Missing period	28	8.8
	Menstrual flow was heavier than usual	100	31.3
	Menstrual flow was lighter than usual	76	23.8
	Periods that lasted longer than 7 days/periods longer than normal	93	29.1
	Periods were shorter than normal	41	12.8
	Periods that are accompanied by pain, cramping, nausea or vomiting	64	20
	Intermenstrual bleeding (vaginal bleeding at any time during the menstrual cycle other than during normal menstruation).	36	11.3
3	For how long the period irregularity lasted? (297 response)		
	1–3 months	183	61.6
	4–6 months	87	29.3
	7–9 months	17	5.7
	More than 9 months	10	3.4
4	Did you visit the gynecologist upon having period irregularity? (359 response)		
	Yes	84	23.5
	No	273	76.5

The duration of menstrual irregularities varied among the vaccinated women, results in Table 2 showed that more than 60% of women had menstrual irregularities lasted from 1 to 3 months, while 29% had it from 4 to 6 months. The menstrual irregularities were a concern for about 24% of the vaccinated women, which made them visiting the gynecologist in this regard (Table 2).

### The COVID-19 vaccine’s effect on the menstrual cycle

To underscore the COVID-19 vaccine effect on menstruation, Pearson correlation analysis was performed to test for the correlation between the menstrual cycle irregularities pre and post vaccination. Results in Table 1 showed that the menstrual irregularities were significantly higher post-vaccination compared to pre-vaccination in women (*r* = 0.199; *p* < 0.001).

Since contraceptives affect the menstrual flow and regularity (34), their correlation with vaccination was assessed and revealed no significant correlation (*r* = 0.009, 0.003) respectively. Remarkably, none of the following health issues (uterine fibroids, thyroid disorders, and osteoporosis) showed a correlation with menstruation pre and post-vaccination (Table 1). Of note, a positive correlation was detected between having ovarian cysts and menstrual irregularities only before vaccination (*r* = 0.183). Whereas the pelvic inflammatory diseases showed a positive correlation only during the vaccination period but not before (*r* = 0.115).

### The effect of the COVID-19 vaccine type on menstrual cycle

The effect of vaccine type on menstrual cycle was assessed. Results in Table 3 showed that 52.6, 51.5, 50.3, and 66.7% of women who received Pfizer–BioNTech, Oxford–AstraZeneca, Sinopharm BBIBP, and Moderna vaccine, respectively, experienced menstrual cycle irregularities during the vaccination period. Further analysis for a possible association between the type of vaccine and the menstrual irregularities using Chi-square test revealed no significant association (Table 3).

**Table 3:** The correlation analysis between the type of COVID-19 vaccine and the menstrual cycle during the vaccination period.

Crosstabulation				
		Did you experience period irregularity during the vaccination period?		Total
		Yes	No	
Which type of the following vaccine did you take?	Pfizer-BioNTech	164	148	312
	%	52.6%	47.4%	100.0%
	AstraZeneca	35	33	68
	%	51.5%	48.5%	100.0%
	Sinopharm	90	89	179
	%	50.3%	49.7%	100.0%
	Moderna	2	1	3
	%	66.7%	33.3%	100.0%
	Else	1	1	2
	%	50.0%	50.0%	100.0%
Total		292	272	564

Chi-Square Test			
	Value	df	Significance (two-sided)
Pearson Chi-Square	0.510^a^	4	0.973
Likelihood Ratio	0.516	4	0.972
Linear-by-Linear Association	0.151	1	0.697
N of Valid Cases	564		

^a^4 cells (40.0%) have expected count less than 5. The minimum expected count is 0.96.

### The effect of vaccine type on the duration of menstrual cycle

Results in Table 4 showed that about 30–34% of vaccines caused menstrual irregularities during the first 3 months of vaccination. The vaccine effect on menstruation declined with time (Table 4). Chi-squared test revealed a significant correlation between the vaccine type and menstrual irregularities duration (*p*-value = 0.004), indicating that Pfizer-BioNTech, AstraZeneca, and Sinopharm vaccines changed the regularity of the menstrual cycle from one to 3 months, whereas Moderna vaccine changed it from one to 6 months (Table 4).

**Table 4:** The correlation analysis between the type of COVID-19 vaccine and the menstrual cycle duration.

Crosstabulation								
			For how long the periperod irregularity lasted (months)?					Total
			No response	1–3	4–6	7–9	More than 9 months	
Which type of the following vaccine did you take?	Pfizer-BioNTech	Count	149	105	47	8	3	312
		%	47.8%	33.7%	15.1%	2.6%	1.0%	100.0%
	AstraZeneca	Count	31	21	10	4	2	68
		%	45.6%	30.9%	14.7%	5.9%	2.9%	100.0%
	Sinopharm	Count	88	55	27	5	4	179
		%	49.2%	30.7%	15.1%	2.8%	2.2%	100.0%
	Moderna	Count	1	1	1	0	0	3
		%	33.3%	33.3%	33.3%	0.0%	0.0%	100.0%
	Else	Count	0	0	1	0	1	2
		%	0.0%	0.0%	50.0%	0.0%	50.0%	100.0%
Total		Count	269	182	86	17	10	564
		%	47.7%	32.3%	15.2%	3.0%	1.8%	100.0%

Chi-Square Test						
	Value	df	Significance (two-sided)			
Pearson Chi-Square	34.914^a^	16	0.004^*^			
Likelihood Ratio	14.218	16	0.582			
N of Valid Cases	564					

^a^13 cells (52.0%) have expected count less than 5. The minimum expected count is 0.04, ^*^ significant.

### Comparison with the VAERS data

**Figure 3:**
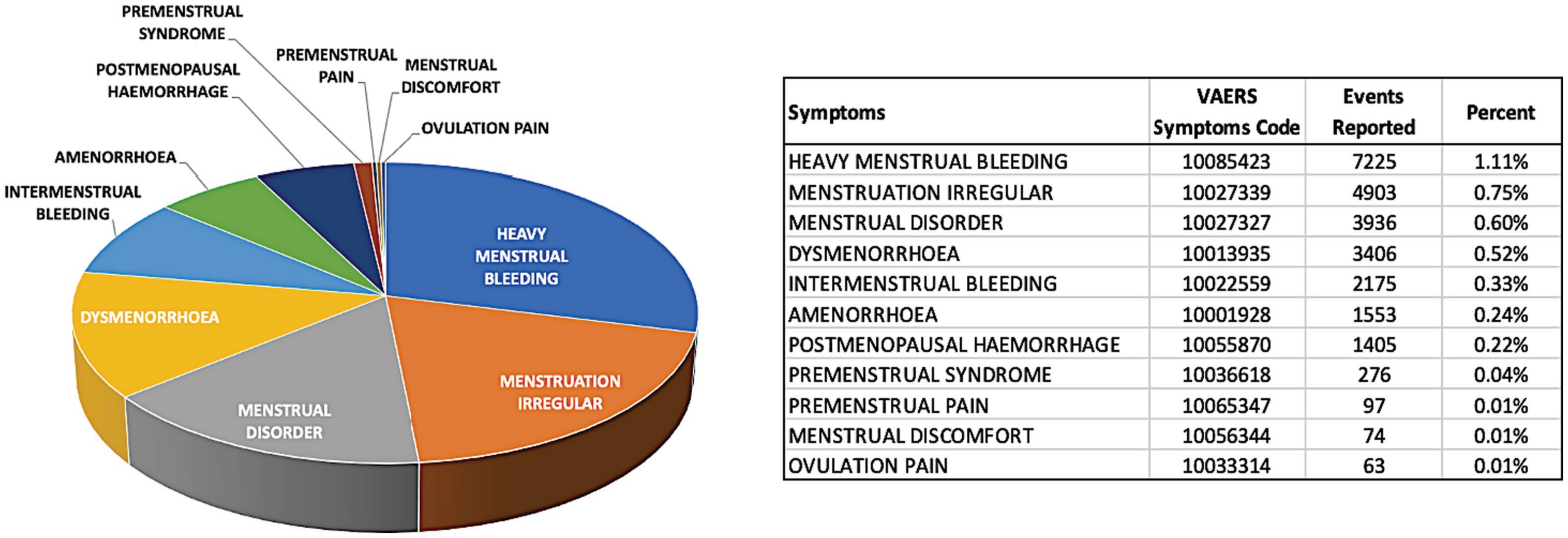
The most frequent menstrual adverse events reported in the VAERS database for COVID-19 vaccines. The pie chart represents visually the share of each of the adverse events related to menstruation-only adverse events. The summary table shows the exact number of reported adverse events and the percent from all adverse event reported for COVID-19 vaccines. VAERS reports processed as of 7th of September 2022 were considered.

Mining the VAERS database for “irregularity menstrual” in response to COVID-19 vaccines indicated that menstruation irregularity constituted 0.75% of all reported adverse events for the COVID-19 vaccines. Other menstrual cycle disturbances were annotated individually and were captured by the manual inspection of all reported adverse event symptoms. Remarkably, the manual search for menstruation-related adverse events such as “heavy menstrual bleeding,” “menstrual disorder” “dysmenorrhoea” (painful period), “intermenstrual bleeding” (bleeding during the menstruation), “amenorrhoea” (absence of menstruation) and “postmenopausal hemorrhage” showed the prevalence of 1.11%, 0.60, 0.52, 0.33, and 0.24%, respectively (Figure 3). Other menstrual cycle related symptoms such as “premenstrual syndrome,” “premenstrual pain,” “menstrual discomfort,” and “ovulation pain” were of lower frequency (i.e., < 0.10%) as shown in Figure 3. This indicates that the database did not really capture the magnanimity of the menstrual irregularities in vaccinated women.

## Discussion

This study was designed to investigate the latest report about COVID-19 vaccine adverse effect on women’s menstruation. Anecdotal reports appeared in the UK Medicine and Healthcare Products Regulatory Agency (MHRA) which indicated that many women were experiencing post-vaccine changes in their menstrual cycles (20, 26). However, these changes were short-lived and are not expected to affect fertility in the vaccinated women (2, 20, 26).

Our results showed that 52% of the vaccinated women experienced post-vaccine menstrual irregularities compared to before. This result was in agreement with other cross-sectional studies, which reported that 50–66% of the vaccinated women experienced menstrual cycle irregularities (21, 26, 35).

Our results are in line with the previous studies showing that the menstrual cycle irregularities were independent of the vaccine type, confirming that all the COVID-19 vaccines may cause menstrual cycle irregularities (21, 26, 35).

About 65% of women in this study reported that the menstrual cycle irregularities were more often after the second vaccine shot. This is in agreement with the previous finding indicating that the menstrual cycle irregularities were slightly higher after the second dose (21). Suggesting additive effect of the vaccine on women. However, another publication reported that the menstrual cycle irregularities were mainly after the first vaccine shot (35). The difference in effect could be attributed to the type of vaccine (mRNA or adenovirus vaccines), or to the immune response of the vaccinated women (20).

Fortunately, the reported menstrual cycle irregularities did not last for a long period of time; most changes were observed and resolved within 3 months following full vaccination regimens. This result is in agreement with previous findings indicating that menstrual cycle changes occurred during the first 2 months after vaccination and totally resolved on their own after that (21, 26, 35).

Vaccinated women reported more than one form of menstrual cycle irregularities. Most of them had more frequent menstrual cycle, while another group reported less frequent menstrual cycle than usual. Additionally, a heavier menstruation than usual was also reported by the vaccinated women. A group of the vaccinated women claimed having menstruation that lasted longer than usual (more than 7 days). This is in agreement with other reports which showed that vaccinated women had longer menstruation with a heavier flow and a wider menstrual frequency than normal periods (21, 35). Taken together, women had distinct responses regarding the nature of the menstrual cycle irregularities, this could be attributed to the time when the woman received the vaccine, which could matches with different phases of woman’s menstrual cycle (follicular or luteal phase), hence the vaccine effect differ from woman to another (21, 26).

Finally, about 52% of the surveyed women claimed menstrual cycle irregularity amid the COVID-19 vaccination, about half of them visited the gynecologist upon having changes in menstruation. Comparing our results to the VAERS data that showed “menstruation irregular” adverse event in only 0.75%. We infer that the menstrual-related adverse events were under reported in the VAERS database for the COVID-19 vaccines compared to other vaccine-induced issues such as myocarditis and pericarditis (16). This could be attributed to many factors; Personal factors, few women tends to seek for medical help upon having problems in menstruation due to lack of awareness or carelessness (36). Social factors, women feel shy to report any health issues related to menstruation, mainly in the low-income and conservative countries (37), a study from Cambodia showed that many young females express fear, shyness, and discomfort during menstruation (37). In addition, there is a lack of studies about menstrual health research in the developing countries (38).

Additionally, such menstrual changes aren’t unique to COVID-19 vaccines; the human papillomavirus (HPV) vaccination was associated with menstrual irregularities (39). This indicates that immune response to viral infections, rather than the viral particle itself, is responsible for inducing this change. However, the exact mechanistic processes linking COVID-19 vaccines to menstrual cycle changes remain elusive. However, some studies tried to link these changes to the nature of the immune response to vaccination indicating some differences between women and women. One study indicated that women developed a higher antibody response and reported various adverse effects in response to COVID-19 vaccination compared to males (40). Other studies reported that COVID-19 vaccination may induce thrombocytopenia which could influence the women’s menstrual cycles (35, 41), leading to loss of endometrial hemostasis and causing heavy bleeding (42). Moreover, the vaccine could affect the production of the female sex hormones, which eventually affect the menstrual cycle stages (23, 43). It should be noted that menstrual cycle irregularities could be attributed to many other factors including viral infection itself (40, 44). In fact, bout 25% of the SARS-CoV-2 infected women had menstrual cycle irregularities upon the pandemic (23).

### Strength and limitations

The strength of this research lies in the flow of the well-structured questionnaire that comprehensively covers the vaccinated women’s demographics, vaccine information, and menstrual cycle status. Additionally, timing of the study reduced the risks of recall bias by conducting the study right after the COVID-19 vaccination process was started in Jordan. This reduced the risk of obtaining inaccurate information provided by the vaccinated women. Furthermore, this study is one of the first studies in Jordan and in the Middle East that discussed menstrual cycle irregularities upon starting the COVID-19 vaccination campaigns. Taken together, the study outcomes enforced the previous findings in the biomedical literature regarding a possible link between COVID-19 vaccines and menstrual cycle changes (21, 26, 45).

However, our research had some limitations. One of the limitations was the women’s willingness to participate in the study and to share their private information regarding their periods, even though the women were informed at the beginning of the study that their information was confidential and the data were collected anonymously. As a result, the sample size was small but it exceeded the minimal statistically-acceptable sample size. This might have created a selection bias preventing the generalizability of the research results. However, this is not uncommon in cross-sectional studies which are often susceptible to the different types of bias according to the nature of the conducted cross-sectional studies. The information bias arises from the women’s knowledge about the different factors that may cause menstrual cycle irregularities including vaccines, and their updated medical records. Further, women’s emotions could be affected by the vaccination or the period changes. Fortunately, our study is free of potential bias since the vaccinated women were approached randomly, and their participation was not subjected to any benefits. Although the research team worked hard to cover all aspects of the research topic, some important information was not covered in the survey like COVID-19 infection information, and some details about menstrual cycle irregularities.

### Implications for practice

The study results advocated the generalizability of the COVID-19 vaccine effect on women’s period regardless of vaccine type. Even though the vaccine effect is temporary, it may affect women’s daily activities and future pregnancy plans. Women need to be informed about the possible changes in the menstrual cycle upon vaccination. It is strongly recommended to investigate the causal effects of vaccines on women’s menstrual cycles at the molecular level. Further investigation on the mechanism of vaccine action on females’ and males’ reproductive systems is highly encouraged. We suggest to address the menstrual cycle irregularities with the COVID-19 vaccine’s possible side effects.

## Conclusion

This study provides evidence that post-COVID-19-vaccine physiological adverse effects during the menstrual cycle were frequently observed in vaccinated women. Fortunately, all studied post-vaccine adverse events concerning menstrual cycle irregularities during menstruations were short-terms and subsided within 3 months on average. Therefore, these adverse events should not be a reason for vaccine hesitancy in young women based on these results.

## Data availability statement

The raw data supporting the conclusions of this article will be made available by the authors, without undue reservation.

## Ethics statement

This study was approved by the Institutional Review Board (IRB) Committee of the Al-Zaytoonah University with no need for writing consent for women’s participation which was optional. Women were informed earlier that all their information are confidential and will be used only for research purposes.

## Author contributions

EA: project administration, conceptualization, investigation, supervision, writing original draft, review, and editing. RH: idea and conceptualization, investigation, writing original draft, review, and editing. AQ: methodology, validation, and writing—review and editing. DS and AA-m: conceptualization, writing original draft, review, and editing. All authors contributed to the article and approved the submitted version.

## Conflict of interest

The authors declare that the research was conducted in the absence of any commercial or financial relationships that could be construed as a potential conflict of interest.

## Publisher’s note

All claims expressed in this article are solely those of the authors and do not necessarily represent those of their affiliated organizations, or those of the publisher, the editors and the reviewers. Any product that may be evaluated in this article, or claim that may be made by its manufacturer, is not guaranteed or endorsed by the publisher.

## Footnotes

^1^https://corona.moh.gov.jo

^2^www.raosoft.com
