# Supplementary material for: A cross-sectional study confirms temporary post-COVID-19 vaccine menstrual irregularity and the associated physiological changes among vaccinated women in Jordan
Source: Front Med (Lausanne). 2023 Oct 6;10:1211283. doi: 10.3389/fmed.2023.1211283 (PMC10587412; doi:10.3389/fmed.2023.1211283)
Supplement: Supplementary file 1 [file Table_1.docx]

**Supplementary data**

**Supplementary Table 1: the period status questions for the COVID-19 vaccinated women in English**

| 1 | **Were you using contraceptives before the vaccination period?** |
| --- | --- |
|  | Yes |
|  | No |
| 2 | **Were you using contraceptives during the vaccination period?** |
|  | Yes |
|  | No |
| 3 | **If your answer was yes for the above question, which type of contraceptives you were using?** |
|  | Combined oral contraceptive Pill |
|  | Intrauterine Device (IUD) |
|  | The Contraceptive Implant |
|  | The Contraceptive Injection |
|  | Others |
| 4 | **Do you have Ovarian cysts?** |
|  | Yes |
|  | No |
| 5 | **Do you have Pelvic inflammatory disease?** |
|  | Yes |
|  | No |
| 6 | **Do you have uterine fibroids?** |
|  | Yes |
|  | No |
| 7 | **Do you have thyroid disorders?** |
|  | Yes |
|  | No |
| 8 | **Do you usually get regular periods (before the COVID19 pandemic)?** |
|  | Yes |
|  | No |
| 9 | **Do you have Osteoporosis?** |
|  | Yes |
|  | No |
| 10 | **Did you experience period irregularity before having the vaccine?** |
|  | Yes |
|  | No |
| 11 | **Did you experience period irregularity during the vaccination period ?** |
|  | Yes |
|  | No |
| 12 | **If your answer was yes for the above question, please describe the kind of irregularity you had by choosing from the following** |
|  | Periods that occur less than 21 days |
|  | Periods that occur more than 35 days apart |
|  | Missing period |
|  | One |
|  | Two |
|  | Three |
|  | More than three |
|  | Menstrual flow was heavier than usual |
|  | Menstrual flow was lighter than usual |
|  | Periods that lasted longer than seven days/periods longer than normal |
|  | Periods were shorter than normal |
|  | Periods that are accompanied by pain, cramping, nausea or vomiting |
|  | Intermenstrual bleeding (vaginal bleeding at any time during the menstrual cycle other than during normal menstruation). |
| 13 | **For how long the period irregularity lasted (months)?** |
|  | 1-3 |
|  | 4-6 |
|  | 7-9 |
|  | More than 9 months |
| 14 | **Have you noticed any change in your premenstrual symptoms (PMS) during the vaccination period (e.g. Bloating, cramping, mood swings, irritability)** |
|  | Yes |
|  | No |
| 15 | **Did you visit the gynecologist upon having period irregularity?** |
|  | Yes |
|  | No |

**Supplementary Table 2: the period status questions for the COVID-19 vaccinated women in Arabic (This table is the Arabic version of the questionnaire; the questions are the same as in the Supplementary Table 1 but in Arabic language because the survey was distributed in Arabic language)**

| **هل كنت تستخدمين وسائل منع الحمل قبل فترة التطعيم؟** | 1 |
| --- | --- |
| نعم |  |
| لا |  |
| **هل كنت تستخدمين وسائل منع الحمل أثناء فترة التطعيم؟** | 2 |
| نعم |  |
| لا |  |
| **إذا كانت إجابتك بنعم على السؤال أعلاه ، فما نوع وسائل منع الحمل التي كنت تستخدمينها؟** | 3 |
| حبوب منع الحمل االتي تؤخذ عن طريق الفم |  |
| جهاز داخل الرحم (اللولب) |  |
| مانع الحمل الهرموني الذي يوضع تحت الجلد |  |
| حقنة منع الحمل |  |
| انواع اخرى |  |
| **هل كنتِ تعانين من تكيسات في المبيض عند اخذكِ المطعوم ؟** | 4 |
| نعم |  |
| لا |  |
| **هل كنتِ تعانين من امرض التهاب الحوض عند اخذكِ المطعوم ؟** | 5 |
| نعم |  |
| لا |  |
| **هل كنتِ تعانين من تليفات في الرحم عند اخذكِ المطعوم ؟** | 6 |
| نعم |  |
| لا |  |
| **هل كنتِ تعانين من اضطرابات في الغدة الدرقية عند اخذكِ المطعوم ؟** | 7 |
| نعم |  |
| لا |  |
| **هل كنتِ تعانين من هشاشة العظام عند اخذكِ المطعوم ؟** | 8 |
| نعم |  |
| لا |  |
| **هل لاحظتِ أي تغيير في أعراض ما قبل الحيض خلال فترة التطعيم, مثل حدوث الانتفاخ ، التشنج ، تقلب المزاج والإنزعاج** | 9 |
| نعم |  |
| لا |  |
|  |  |
| **هل عانيتِ من عدم انتظام الدورة الشهرية قبل أخذ المطعوم ؟** | 10 |
| نعم |  |
| لا |  |
| **هل عانيتِ من عدم انتظام الدورة الشهرية خلال فترة التطعيم؟** | 11 |
| نعم |  |
| لا |  |
| **إذا كانت إجابتكِ بنعم على السؤال أعلاه ,الرجاء اكمال الاسئله التاليه**** |  |
| **متى لاحظتِ عدم الانتظام في الدورة الشهرية (يمكن اختيار أكثر من خيار واحد** | 12 |
| بعد أخذ الجرعة الاولى |  |
| بعد أخذ الجرعة الثانية |  |
| بعد أخذ الجرعة الثالثة |  |
| **الرجاء وصف نوع عدم الانتظام في الدورة الشهرية الذي عانيتِ منه نتيجة المطعوم** | 13 |
| **(يمكن اختيار أكثر من خيار واحد** |  |
| الفتره الزمنيه بين الدوره والتي تليها اقل من 21 يومًا |  |
| الفتره الزمنيه بين الدوره والتي تليها اكثر من 35 يومًا |  |
| غياب الدوره الشهريه |  |
| تدفق الدورة الشهرية أكثر من المعتاد |  |
| تدفق الدورة الشهرية أخف من المعتاد |  |
| استمرارالدوره الشهريه لأكثر من سبعة أيام / فترات أطول من المعتاد |  |
| استمرارالدوره الشهريه لأقل من سبعة أيام / فترات أقصر من المعتاد |  |
| صاحب الدوره الشهريه ألم أو تشنج أو غثيان أو قيء غير مسبوق |  |
| نزيف ما بين فترات الحيض (نزيف مهبلي في أي وقت بين الدورة الشهرية والتي تليها بخلاف الدورة الشهرية العادية |  |
| **ما هي مدة عدم الانتظام في الدورة** الشهرية )**بالشهور)؟** | 14 |
| 1-3 |  |
| 4-6 |  |
| 7-9 |  |
| أكثر من 9 شهور |  |
| **هل قمتِ بزيارة طبيب النساء عند حدوث عدم الانتظام في الدورة الشهرية خلال فترة اخذ المطعوم؟** | 15 |
| نعم |  |
| لا |  |
